# In Situ Self-Growth of a ZnO Nanorod Array on Nonwoven Fabrics for Empowering Superhydrophobic and Antibacterial Features

**DOI:** 10.3390/molecules29122916

**Published:** 2024-06-19

**Authors:** Xiaoqi Yuan, Binghui Liu, Aili Yang, Peng Zhang, Wenjie Li, Yueyu Su

**Affiliations:** 1 School of Environmental Science and Engineering, Xiamen University of Technology, Xiamen 361024, China; 2 Institute for Energy, Environment and Sustainable Communities, University of Regina, Regina, SK S4S 0A2, Canada

**Keywords:** ZnO nanoarray, modified nonwoven fabric, response surface methodology, hydrophobicity, antibacterial capacity

## Abstract

ZnO nanorod nonwoven fabrics (ZNRN) were developed through hydrothermal synthesis to facilitate the prevention of the transmission of respiratory pathogens. The superhydrophobicity and antibacterial properties of ZNRN were improved through the response surface methodology. The synthesized material exhibited significant water repellency, indicated by a water contact angle of 163.9°, and thus demonstrated antibacterial rates of 91.8% for Escherichia coli (*E. coli*) and 79.75% for Staphylococcus aureus (*S. aureus*). This indicated that *E. coli* with thinner peptidoglycan may be more easily killed than *S. aureus*. This study identified significant effects of synthesis conditions on the antibacterial effectiveness, with comprehensive multivariate analyses elucidating the underlying correlations. In addition, the ZnO nanorod structure of ZNRN was characterized through SEM and XRD analyses. It endows the properties of superhydrophobicity (thus preventing bacteria from adhering to the ZNRN surface) and antibacterial capacity (thus damaging cells through the puncturing of these nanorods). Consequently, the alignment of two such features is desired to help support the development of personal protective equipment, which assists in avoiding the spread of respiratory infections.

## 1. Introduction

Based on the report of the World Health Organization, influenza is a seasonal infection that can annually cause an estimated 1 billion infections and between 290,000 and 650,000 deaths worldwide [1]. This severe consequence worsens when influenza aligns with another respiratory virus, such as COVID-19, resulting in a worldwide pandemic. Such respiratory viruses are mainly spread from person to person through droplets [2]. Therefore, the development of personal protective equipment with antibacterial and hydrophobic features to prevent the daily spread of the virus is desired.

Recently, micro-nano structures on the surfaces of insects with features such as superhydrophobicity, self-cleaning, and directional wetting provided inspiration [3]. The unique antibacterial properties of cicadas and dragonflies are attributed to needle-like nanorods on their wing surfaces [4]. In addition, studies reported that such nanorods caused more damage to bacteria with weaker stretching ability and stronger adhesion [5,6]. Contact between the cell wall and nanorods led to cell membrane damage, resulting in cell death [7]. In addition, genomic and proteomic studies further identified that the proteins related to the cell membrane and DNA expressed an intense stress response even when the cells did not exhibit bacterial cleavage [8]. Based on this insect structure, multiple antibacterial materials, such as nanomaterials, polymers, and their composites, have been developed to investigate the effects of nano-column sharpness and spacing on antibacterial capacity for various bacteria with varying stretching and adhesion capabilities [9,10]. Dopamine was employed as a binder to coat a layer of nano-copper on wood surfaces to fabricate wood@PDA-Cu-F material [11]. This material exhibited a contact angle of 155.7° and antibacterial performance against both *E. coli* and *S. aureus*. In addition, plasma etching constructed a nanostructured surface with 200 nm-long nano-pillars, resulting in a bactericidal rate of 30% [12,13,14]. is indicated that the nanostructure-associated synthesis conditions were critical to material properties, and the effects of relevant synthesis conditions and their interactions were complicated in the fabrication of high-performance materials.

The application of antibacterial coatings in nonwoven fabrics has attracted widespread attention. For example, previous studies have shown that silver nanoparticles were a common antibacterial coating material and exhibited good antibacterial effects on nonwoven fabrics made of cellulose substrates and polymers. However, even at low doses, silver nanoparticles exhibited high toxicity to normal cells, which limited their application in personal protective equipment [15]. Nano-graphene coatings, as a new type of nanomaterial, also had certain antibacterial properties, but their synthesis was complex, requiring sophisticated equipment such as electrospinning and rolling machines or the addition of complex adhesives for the application to nonwoven fabrics. This not only resulted in high synthesis costs but also required advanced technology [16,17]. In contrast, ZNRN synthesis is simple and low-cost, accomplished solely through a hydrothermal reaction, and exhibits good antibacterial performance [18]. The high aspect ratio and sharp edge of ZnO nanorods enabled them to physically interact with bacterial cell membranes, which may lead to cell structural damage and cell death. This mechanical antibacterial activity is particularly effective for different types of bacteria and can destroy bacteria, including drug-resistant strains [19]. In addition, ZnO exhibits excellent chemical stability and safety [20], making it an ideal material for the manufacture of personal protective equipment for long-term use.

However, there has been no report on growing nanorods on nonwoven fibers with superhydrophobic and antibacterial features that prevent bacteria from adhering and growing. Furthermore, the interactions among synthesis variables are still not clear, which is crucial to identify suitable variables for desired bactericidal-antibacterial effects of fabricated materials, and thus, it is critical to reveal associated mechanisms.

Therefore, the objective of this study is to develop ZnO nanorod nonwoven fabrics (ZNRN) with both antibacterial and superhydrophobic features to help fabricate personal protective equipment. In detail, it entails the (1) in situ growth of ZNRN through the hydrothermal method; (2) identification of synthesis variables (Zn(NO_3_)_2_ concentration, HMTA concentration, temperature, synthesis duration) for the desired hydrophobic and antibacterial properties through the response surface methodology; (3) analysis of multi-factor interactive effects on antibacterial capability and hydrophobicity through multivariate factorial analysis; (4) characterization of ZNRN for revealing associated mechanisms through water contact angle, SEM, XRD, XPS, and EDS analyses.

## 2. Results and Discussion

### 2.1. Material Characterization

**Figure 1:**
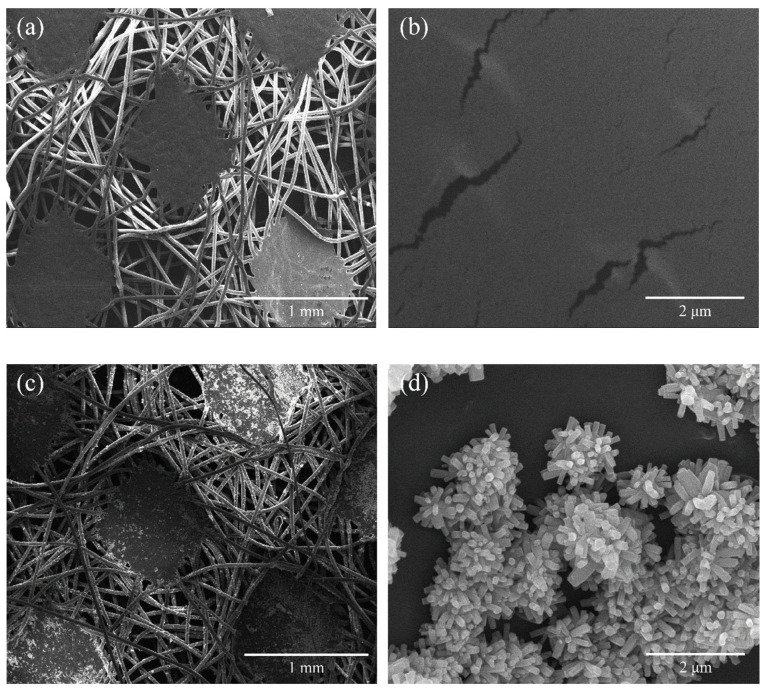
SEM pictures from pristine and modified nonwoven fabrics. (**a**) pristine fabric and (**b**) associated zoom-in one; (**c**) modified fabric, and (**d**) associated zoom-in one.

The pristine nonwoven fabrics are depicted in Figure 1a,b, while ZNRN samples are shown in Figure 1c,d. Notably, patches of nano-ZnO crystals were observed attached to the fiber surface, appearing in a connected and agglomerated state (Figure 1c. A conical rod-shaped structure was further observed in Figure 1d. The desired outcome of the conical nanoarray ZnO attached to the nonwoven fabric is its ability to physically eliminate adhering microbes by mechanically lysing bacterial cells without triggering potential antibacterial resistance [21,22,23].

**Figure 2:**
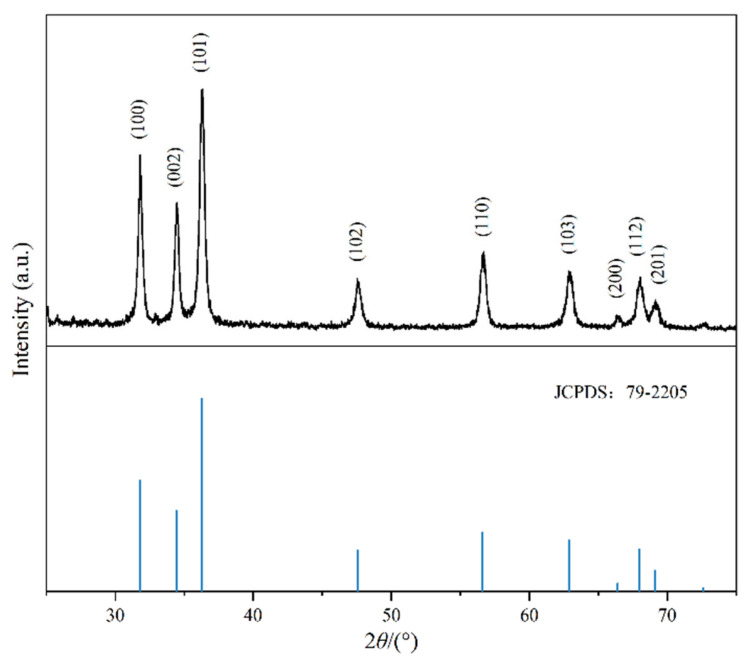
XRD pattern of ZNRN.

The X-ray diffraction spectrum of the ZNRN sample is shown in Figure 2, revealing distinct diffraction peaks at various 2θ degrees: 31.765°; 34.418°; 36.250°; 47.534°; 56.589°; 62.850°; 66.369°; 67.940°; and 69.078°. These peaks were associated with the (100), (002), (101), (102), (110), (103), (200), (112), and (201) planes, respectively. The observed diffraction pattern was consistent with the characteristic X-ray diffraction peaks and lattice spacings of ZnO crystals with a hexagonal wurtzite structure (JCPDS: 79-2205) [24]. This alignment strongly suggests the successful loading of ZnO onto the surface of the nonwoven fabric. The growth process of nano ZnO was divided into two parts: the nucleation process and the secondary growth process. The first step involved the formation of ZnO seed nuclei, and the second step was the continuous growth toward specific crystal faces based on the crystal nucleus. Moreover, the synthesis duration determined the completeness of the ZnO crystal growth [25,26]. In addition, the EDS of ZNRN is shown in Figure S1a–f, which confirmed that ZNRN also contained Zn and O elements in addition to the C elements existing in the original nonwoven fabric. Figure S1b shows the corresponding EDS spectra of Zn elements. The XPS diagram of the modified nonwoven fabric is shown in Figure S2. Figure S2a shows the total XPS spectrum of ZNRN (A) and the original unmodified nonwoven fabric (C). It could be seen from the diagram that the modified nonwoven fabric obviously had more obvious Zn peaks than the original unmodified nonwoven fabric. Figure S2b is a partial peak fitting diagram of Zn2p. It was shown that the spectra at 1022.2 eV and 1045.2 eV corresponded to Zn2p3/2 and Zn2p1/2 of Zn in ZnO. Therefore, nano-zinc oxide was successfully loaded onto the nonwoven fabric.

**Figure 3:**
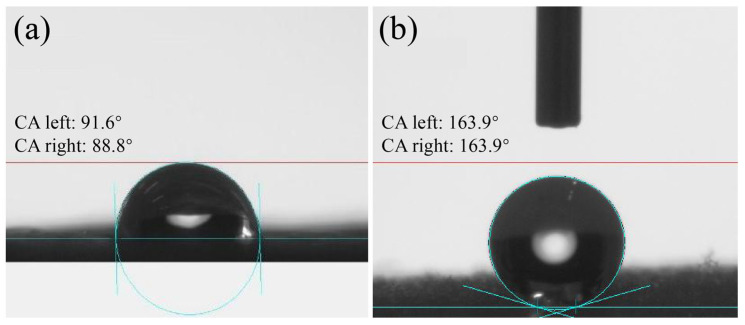
Pictures of water-contact angle. (**a**) pristine fabric and (**b**) ZNRN.

As shown in Figure 3, the contact angle between the original and ZNRN fabrics changed significantly. The contact angle for the pristine fabric ranged from 91.6° to 88.8°. In contrast, the contact angle of the ZNRN fabric reached 163.9°, indicating a superhydrophobic surface (contact angle greater than 150°) [27]. This superhydrophobic surface is associated with several advantages, including a reduction in protein adsorption [28], prevention of biological blockage [29], and intrinsic antibacterial activity against adhering bacteria [30]. Consequently, such a superhydrophobic surface is effective in preventing the adherence of respiratory infection droplets from coughs or sneezes. Therefore, the superhydrophobic ZNRN fabric was endowed with the capability to not only diminish the adhesive capacity of microorganisms but also enhance self-cleaning and antibacterial properties.

### 2.2. Determination of Optimal Conditions through Response Surface Methodology

Depending on the results of the response surface experimental design (see Supplementary Materials Table S1), ZNRN antibacterial models for the inhibition rates Y_1_ of *E. coli* and Y_2_ of *S. aureus* were developed through multivariate regression, respectively:

$$Y_{1}=93.75-8.24A+2.79B+0.6475C+10.86D+6.55\mathrm{AB}+18.85\mathrm{AC}+1.86\mathrm{AD}+\;15.29\mathrm{BC}+11.43\mathrm{BD}-13.88\mathrm{CD}\;-6.40A^{2}-18.61B^{2}-19.89C^{2}-5.37D^{2}$$

$$Y_{2}=81.74-1.36A+7.57B-1.54C-4.43D-0.61\mathrm{AB}-1.87\mathrm{AC}+4.85\mathrm{AD}-2.12\mathrm{BC}+1.61\mathrm{BD}-0.63D\;-5.80A^{2}-4.82B^{2}-6.06C^{2}-2.86D^{2}$$

**Figure 4:**
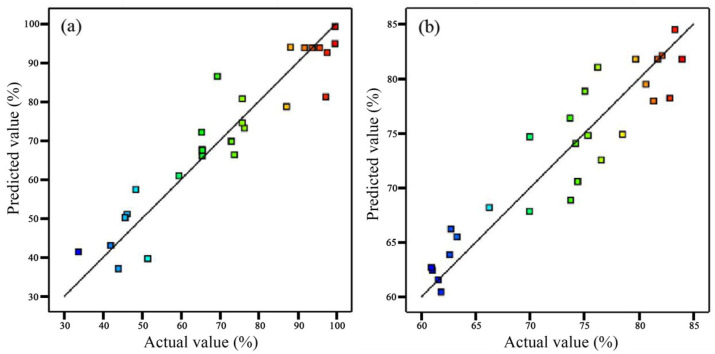
Evaluation of regression models of ZNRN antibacterial capabilities to inhibit (**a**) *E. coli* and (**b**) *S. aureus*.

According to the statistical information of the models (see Supplementary Materials Table S2), the adjusted determination coefficients *R^2^* for these two models were 0.8832 and 0.8697 (*p* < 0.01), respectively, indicating that the models can explain 88.32% and 86.97% of the changes in response values. The *p*-values for the lack of fit were 0.0296 and 0.1970 (*p* > 0.05), respectively. The insignificance of the lack of fit indicates that the model fit was relatively reliable. The coefficients of variation *C.V.* for the models were 14.23% and 5.76%, respectively. The signal-to-noise ratio indexes (*Adeq*. Precision) were 8.1907 and 7.6546, respectively. These suggested that both models had a high degree of fit and reliability, and the regression results were good [31,32]. In addition, the experimental values of these two models for Y_1_ of *E. coli* and Y_2_ of *S. aureus* were evenly and closely distributed on both sides of the predicted fitting line (Figure 4), indicating the high fit and accuracy of the experimental values. 

Based on the established models, the optimal antibacterial rates of ZNRN within the definitional domain for each factor were 91.80% and 79.75% for *E. coli* and *S. aureus*, respectively. These optimal results aligned with a ZNRN synthesis condition featuring a Zn(NO_3_)_2_ concentration of 0.072 mol/L, a hexamethylenetetramine (HMTA) concentration of 0.091 mol/L, a heating temperature of 90.069 °C, and a synthesis duration of 2.797 h. It was noteworthy that the optimized antibacterial effect on *E. coli* was significantly superior to that on *S. aureus*. Previous studies suggested that nanoarrays could penetrate *S. aureus*, albeit at a lower frequency, compared to *E. coli* [33,34]. Additionally, the deformation of the *S. aureus* capsule in the nanoarrays may be attributed to an increase in the thickness of the peptidoglycan layer, requiring higher hardness and expansion pressure [35]. In addition, the force required to penetrate the cell wall of *S. aureus* was four times that of *E. coli*. Therefore, ZNRN would need a longer contact time with Gram-positive bacteria to reach a similar antibacterial performance for Gram-negative bacteria.

The significant effects of factors and their interactions (*p* < 0.05) on ZNRN bactericide capability are shown in Table 1. For *E. coli*, the antibacterial rate of ZNRN, zinc nitrate concentration (A), and synthesis duration (D) were significant one-order factors, while AC, BC, CD, and BD were significant interactions. The *F* value reflected the intensity of the effect, and thus, the associated intensities of individual factors were in the following order: synthesis duration (D) > zinc nitrate concentration (A) > HMTA concentration (B) > heating temperature (C). On the other hand, HMTA concentration (B) and synthesis duration (D) were significant one-order factors for the ZNRN antibacterial rate of *S. aureus*, while AD were significant interactions. Associated intensities of individual factors were in the following order: HMTA concentration (B) > synthesis duration (D) > heating temperature (C) > zinc nitrate concentration (A).

**Figure 5:**
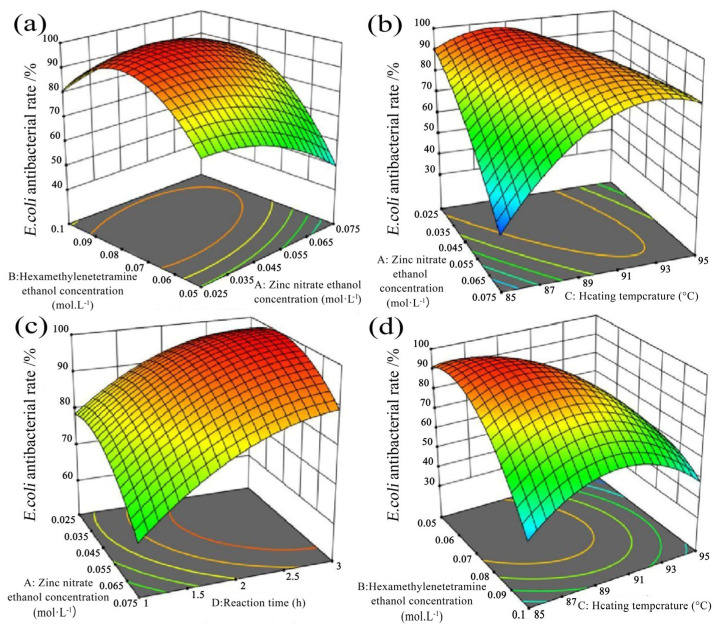
Contour patterns of two-factor combination effects on *E. coli* antibacterial rate of ZNRN. (**a**) interaction of HMTA concentration × Zn(NO_3_)_2_ concentration, (**b**) interaction of Zn(NO_3_)_2_ concentration × temperature, (**c**) interaction of Zn(NO_3_)_2_ concentration × reaction time, and (**d**) interaction of HMTA concentration × temperature.

**Figure 6:**
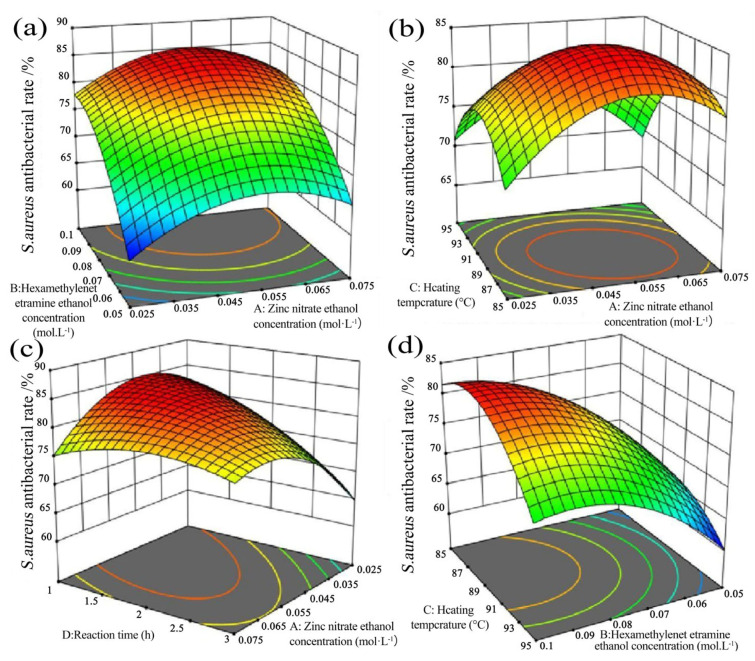
Contour patterns of two-factor combination effects on *S. aureus* antibacterial rate of ZNRN. (**a**) interaction of HMTA concentration × Zn(NO_3_)_2_ concentration, (**b**) interaction of Zn(NO_3_)_2_ concentration × temperature, (**c**) interaction of Zn(NO_3_)_2_ concentration × reaction time, and (**d**) interaction of HMTA concentration × temperature.

The two-way interactions of ZNRN on the antibacterial rate of *E. coli* and *S. aureus* were further exhibited through response surfaces shown in Figure 5 and Figure 6. Typically, the different shapes of the contour maps represent different interactions between variables [36,37]. Greater curvature of the response surface corresponds to a stronger interaction between the two variables [38,39]. Additionally, a larger slope of the response surface in the direction of a specific variable (parallel to the axis of the variable) amplifies its impact on the response value [40]. Simultaneously, colors represented response values, with warmer colors indicating higher response values. The lines connected the response values for different levels of interaction between the two factors.

Under the conditions of a heating temperature of 90.5 °C and a synthesis duration of 2.5 h, with the concentration of zinc nitrate held constant, the antibacterial rate against *E. coli* initially increased and then decreased with an increase in the HMTA concentration (Figure 5a). Conversely, when the concentration of HMTA was held constant, the antibacterial rate against *E. coli* gradually decreased as the concentration of zinc nitrate increased. Under the conditions of a hexamethylenetetramine (HTMA) concentration of 0.078 mol/L, with a constant Zn(NO_3_)_2_ concentration and a synthesis duration of 1.96 h, the antibacterial rate against *E. coli* exhibited an initial increase followed by a decrease with the rise in heating temperature (Figure 5b). On the other hand, with a constant heating temperature, the antibacterial rate against *E. coli* rapidly decreased as the zinc nitrate concentration increased. Under a condition of an HMTA concentration of 0.078 mol/L and constant Zn(NO_3_)_2_ concentration at a heating temperature of 90.5 °C, the antibacterial rate against *E. coli* demonstrated a rapid increase with the extension of synthesis duration (Figure 5c). Conversely, when the synthesis duration was held constant, the antibacterial rate against *E. coli* gradually decreased with the increase in the zinc nitrate concentration.

While under the condition of a zinc nitrate concentration of 0.025 mol/L and a synthesis duration of 1.72 h, with a constant concentration of HMTA, the antibacterial rate against *E. coli* slowly increased with rising heating temperatures and then rapidly decreased (Figure 5d). Conversely, with a constant heating temperature, the antibacterial rate against *E. coli* quickly decreased with the increase in the HMTA concentration.

In Figure 6b, with a heating temperature of 88.4 °C, a synthesis duration of 2 h, and a constant zinc nitrate concentration, the antibacterial rate against *S. aureus* rapidly increased with an increase in the HMTA concentration. Conversely, when the HMTA concentration was constant, the antibacterial rate against *S. aureus* initially increased and then gradually decreased with an increase in the zinc nitrate concentration. In Figure 6b, under the condition of an HMTA concentration of 0.082 mol/L and a constant zinc nitrate concentration with a synthesis duration of 1.98 h, the antibacterial rate against *S. aureus* initially increased and then decreased with rising heating temperatures. Similarly, when the heating temperature was constant, the antibacterial rate against *S. aureus* initially increased and then decreased with an increase in the zinc nitrate concentration. In Figure 6c, with an HMTA concentration of 0.082 mol/L and a constant zinc nitrate concentration at a heating temperature of 89.8 °C, the antibacterial rate against *S. aureus* decreased with an increase in synthesis duration. Conversely, when the synthesis duration was constant, the antibacterial rate against *S. aureus* initially increased and then gradually decreased with an increase in the zinc nitrate concentration. In Figure 6d, with the same HMTA concentration and a zinc nitrate value of 0.065 mol/L, a constant HMTA concentration, and a synthesis duration of 2.42 h, the antibacterial rate against *S. aureus* quickly decreased with rising heating temperatures. Conversely, when the heating temperature was constant, the antibacterial rate against *S. aureus* increased with an increase in the HMTA concentration.

### 2.3. Analysis of Antibacterial and Superhydrophobic Effects of Single Factors

The purpose of a single-factor experiment is to experiment by varying a single variable, observing the influence of that variable on the research metrics, and thereby exploring the underlying patterns. Even if the variable does not exhibit a systematic impact on the research metrics, valuable insights can be gained from the experimental results, including the range of the variable where significant changes in the research metrics occur.

**Figure 7:**
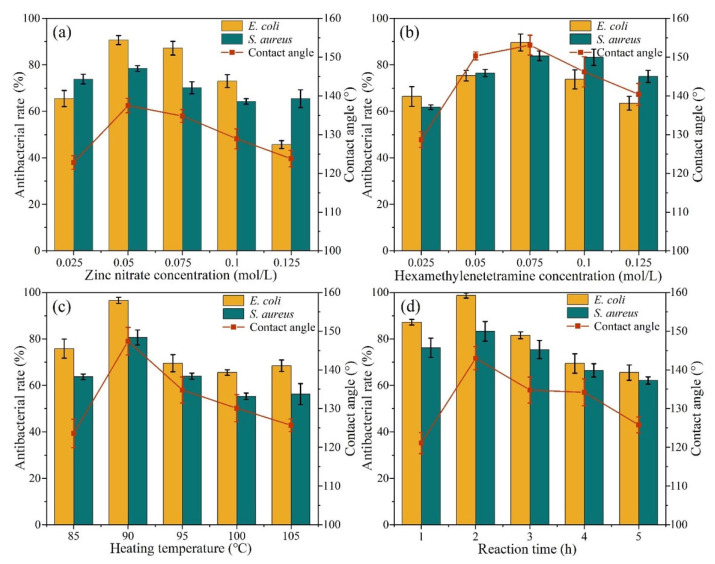
Effects of individual factors on antibacterial capability and hydrophobicity. (**a**) Zn(NO_3_)_2_ concentration, (**b**) HMTA concentration, (**c**) heating temperature, and (**d**) reaction time.

Antibacterial properties and hydrophobicity of ZNRN involving multiple factors are shown in Figure 7. The hydrophobicity significantly increased with an HTMA concentration between 0.025 and 0.075 mol/L. It was observed that the hydrophobicity of the nonwoven fabric reached a maximum at 153° under an HTMA concentration of 0.075 mol/L. It was also noted that HTMA becomes insoluble in an ethanol solution after reaching a concentration of 0.1 mol/L, inhibiting the hydrophobicity and antibacterial properties of the nonwoven fabric. In previous studies, it was observed that the concentration of HTMA uniquely influences the anisotropic growth of nano ZnO [41]. When the concentration of HTMA increased, the growth of ZnO nanorods became denser, and the hexagonal shape was modified to a circular shape. This adverse change also reduced the destruction of microorganism cell membranes [42]. In addition, as shown in Figure 7, the antibacterial rate of *E. coli* was generally greater than that of *S. aureus* because of its different structure. Previous studies have shown that Gram-positive bacteria, such as *S. aureus*, have thicker peptidoglycan cell walls than Gram-negative bacteria [43].

### 2.4. Discussion

**Figure 8:**
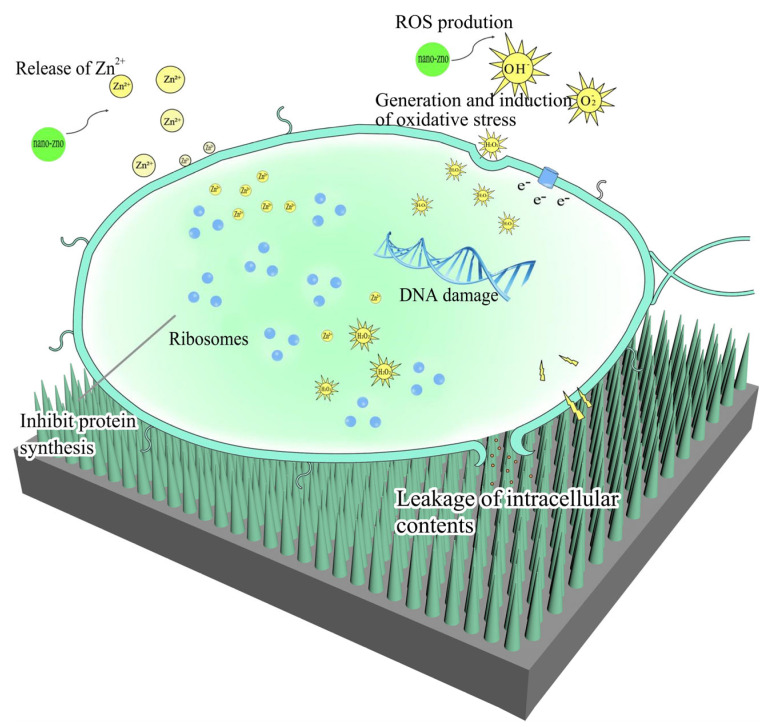
ZNRN antibacterial mechanisms. (1) oxidative stress of reactive oxygen species (ROS), (2) cell internalization of dissolved Zn^2+^ ion, and (3) punctures of cell wall/membrane damaged by nano-array.

The developed ZNRN exhibited excellent superhydrophobicity with a contact angle of 163.9°, coupled with significant bactericidal properties (91.80% for *E. coli* and 79.75% for *S. aureus*). These properties were attributed to the presence of the ZnO nano-pillar array, making ZNRN a promising candidate for producing personal protective equipment in the context of respiratory infections. The bactericidal mechanisms of ZNRN is illustrated in Figure 8 and Figures S3–S5 in Supplementary Materials. When zinc oxide nanoparticles come into contact with bacteria, the nanostructured arrays of zinc oxide can physically puncture the bacterial cells, further disrupting membrane integrity and leading to leakage of cellular contents [44]. In addition, released Zn ions from the ZnO nano-pillar array also bind with the negatively charged bacterial membrane, causing it to be broken. Subsequently, these ions can further enter the bacterial cell to inhibit enzyme function and deactivate proteins and DNA, thus terminating bacterial metabolism [45]. In addition, zinc oxide produces reactive oxygen species (ROS) when exposed to appropriate light [46]. Such ROS react with organic biomolecules, such as lipids, carbohydrates, proteins, etc., in bacterial cells, causing oxidative degradation of the cell membrane and even damage to relevant tissues [47].

On the other hand, the formation of nanorod arrays enhanced ZNRN hydrophobicity. Water droplets were formed from the cohesion among water molecules, which rolled off the surface of the nonwoven fabric instead of spreading on the surface. In this way, the bacteria encapsulated in these droplets left the ZNRN surface, suspending them from growing and reproducing on the surface. Hence, the developed ZNRN could not only lead to bacterial death but also facilitate self-cleaning removal of damaged cells from the superhydrophobic surface.

## 3. Materials and Methods

### 3.1. Process of Material Synthesis

**Figure 9:**
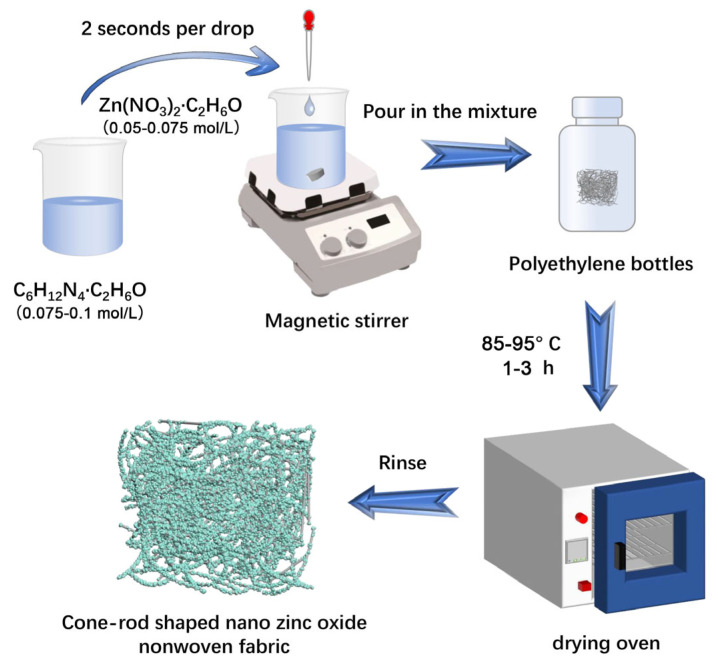
Process of ZNRN fabrication.

Polypropylene nonwoven fabric (Jialianda Nonwoven Fabric Co., Ltd., Dongguan, China) was cut into pieces with the size of 2 cm × 2 cm. These fabrics were sequentially washed in a mixture of petroleum ether, anhydrous ethanol, and deionized water for 1 h in an ultrasonic cleaner. The cleaned fabrics were dried in an oven at 60 °C for 24 h and then stored and prepared for later use. Different amounts of hexamethylenetetramine and zinc nitrate chemicals were added separately into a beaker containing 50 mL of anhydrous ethanol. They were stirred with a glass rod until dissolved, thereby preparing hexamethylenetetramine ethanol solution (0.075–0.1 mol/L, C6H12N4, Shanghai Ep Biotech Co., Ltd., Shanghai, China) and zinc nitrate ethanol solution (0.05–0.075 mol/L, Zn(NO_3_)_2_, Tianjin Guangfu Technology Development Co., Ltd., Tianjin, China). The hexamethylenetetramine ethanol solution was then added dropwise into the zinc nitrate ethanol solution at a rate of 2 drops per second, followed by stirring with a magnetic stirrer for 5 min [48]. When a white suspension was formed in the beaker, the suspension was transferred into a polyethylene bottle containing the prepared fabrics. Such a bottle was sealed and heated in an air-drying oven at 85–95 °C and heated for 1–3 h (The specific values for the four factors, namely hexamethylenetetramine ethanol solution, zinc nitrate ethanol solution, temperature, and reaction time, were referenced from Table S1). After this hydrothermal procedure, the fabrics were washed repeatedly until the ZnO residue was removed by deionized water. These cleaning fabrics were further dried at 60 °C, and ZnO nanorod nonwoven fabrics (ZNRN) were fabricated [49]. The process is illustrated in Figure 9.

### 3.2. Examination of Antibacterial Properties

Gram-positive bacteria (i.e., *S. aureus*) and Gram-negative bacteria (i.e., *E. coli*) were chosen as target bacteria to investigate the antibacterial capacity of ZNRN through the AATCC-100 antibacterial method (agar diffusion method). Briefly, ZNRN and pristine fabrics (i.e., control) were cut into 2 × 2 cm squares, and then these squares were sterilized at 121 °C in a high-pressure sterilizer for 15 min. The sterilized samples were placed in multiple Petri dishes, and 50 μL of bacterial fluid with a magnitude of 5 × 10^7^ CFU/mL was added to individual samples. After 24-h of incubation (at 37 °C), individual infected fabric samples were put into sealed vials with 10 mL phosphate buffer solution, followed by 5-min vortex shaking. One milliliter of bacterial suspension from each vial was transferred to an LB agar plate for a 24 h incubation (at 37 °C). The number of bacteria in each plate was counted after incubation, and the associated antibacterial rate (R) was calculated as,

(1) $$R=\frac{M_{1}-{\;M}_{2}}{M_{1}}\times 100\%$$

M_1_ and M_2_ are the number of bacteria on control and ZNRN samples, respectively.

### 3.3. Characterization of Physicochemical Properties

The surface morphology and elemental composition of ZNRN and control samples were studied by SEM (SEM, Inspect F50, Bruker AXS, Karlsruhe, Germany). The crystallinity of ZNRN was examined through an X-ray diffractometer (XRD, D8 ADVANCE, Bruker, Karlsruhe, Germany). The water contact angle of ZNRN and control samples was measured through a contact angle analyzer (OCA15EC, DataPhysics Instruments GmbH, Filderstadt, Germany). X-ray photoelectron spectroscopy was used to analyze the energy spectrum (XPS, 250Xi, Thermo scientific, Waltham, MA, USA). The morphology of the bacteria was studied by emission scanning electron microscopy (SEM, Hitachi SU8010, CIQTEK, Tokyp, Japan). The morphology of bacteria was studied by transmission electron microscopy (TEM, JEM-1230, JEOL, Akishima, Japan). The ROS levels of the materials were studied by flow cytometer (FC, cytoflex, Beckman, LA, USA).

### 3.4. Identification of Synthetic Factors

The antibacterial capacity of ZNRN depends on the associated physicochemical properties, which are affected by multiple synthetic factors, particularly in their interactions. In order to reveal such complicated effects of multiple factors and their interactions, a four-factor (Zn(NO_3_)_2_ concentration, HMTA concentration, temperature, and hydrothermal duration) 3-level (low, medium, and high) factorial analysis was designed based on the response surface methodology (Table 2). Significant main and interactive factors were identified based on F-test analysis (*p* < 0.05). The modeling of the antibacterial rates of *E. coli* and *S. aureus* on ZNRN was further undertaken based on multivariate regression analysis. In addition, the desired synthetic factors of ZNRN for the best antibacterial performance were conducted based on the developed models.

In order to further explore the main factors affecting the destruction of *E. coli* and *S. aureus* and hydrophobicity, multiple investigations were undertaken in a range of individual factors (i.e., (0.025–0.075) mol/L of Zn(NO_3_)_2_, (0.050–0.1) mol/L of HMTA, (85–95) °C of temperature, and (1–3) h of synthesis duration).

### 3.5. Quality Control

The reagents involved in this study were chemical grade (>99%), and all tests were replicated three times for quality assurance and control. The statistical analyses and related testing were conducted under the significance level of 0.05 (*p* < 0.05).

## 4. Conclusions

In this study, ZnO nanorod nonwoven fabrics (ZNRN) with dual functions in terms of superhydrophobic and antibacterial features were fabricated through hydrothermal synthesis. The synthesis variables of the desired ZNRN were 0.072 mol/L Zn(NO_3_)_2_, 0.091 mol/L HMTA, 90.07 °C, and 2.8 h. The water contact angle of this desired ZNRN was 163.9°, and the associated antibacterial performance against *E. coli* and *S. aureus* was 91.8% and 79.75%, respectively. For *E. coli,* the antibacterial rate of ZNRN, Zn(NO_3_)_2_ concentration, and synthesis duration were the main significant factors, while the interactions of Zn(NO_3_)_2_ × temperature, HMTA × temperature, temperature × duration, and HMTA × duration were significant. On the other hand, the HMTA concentration and duration were the main significant factors for the ZNRN antibacterial rate of *S. aureus*, while the interactive effect of Zn(NO_3_)_2_ × duration was significant on the antibacterial rate of *S. aureus*. SEM, XRD, XPS, and EDS analyses indicated that the ZnO nanorod array was grown on nonwoven fabrics. Such an array provided a unique structure, such as the skin surface of insects, which could facilitate droplets to drop from the skin through the lotus effect, reducing the solid–liquid surface energy. Contaminants (e.g., dirt, bacteria, viruses, etc.) on such a unique surface were further picked up by water droplets, causing a self-cleaning effect. Furthermore, negatively charged bacterial cells were attracted to the ZNRN surface and were further destroyed through physical puncture of the fabricated array, leading to the leakage of intracellular contents. The combination of superhydrophobic and antibacterial features endowed the ZNRN with a robust capability to avoid the adhesion of bacteria-containing droplets and further demote bacterial growth. Consequently, the developed ZNRN with superhydrophobic and antibacterial capacities could be applied to the fabrication of personal protective equipment (i.e., masks and coats) to prevent infection of respiratory pathogens.

## Figures and Tables

**Table 1:** Response surface modeling information for ZNRN antibacterial capability.

Source	*E. coli* Antibacterial Rate		Significance	*S. aureus* Antibacterial Rate		Significance
	*F*-Value	*p*-Value		*F*-Value	*p*-Value	
Model	6.48	0.0013	**	5.72	0.0022	**
A—Zn(NO_3_)_2_	7.89	0.0158	*	1.26	0.2837	-
B—HMTA	0.9064	0.3599	-	38.85	< 0.0001	**
C—Temperature	0.0487	0.8290	-	1.60	0.2297	-
D—Duration	13.70	0.0030	**	13.32	0.0033	**
AB	1.66	0.2214	-	0.0827	0.7786	-
AC	13.76	0.0030	**	0.7918	0.3910	-
AD	0.1344	0.7203	-	5.31	0.0399	*
BC	9.82	0.0086	**	1.01	0.3336	-
BD	5.06	0.0440	*	0.5836	0.4597	-
CD	7.46	0.0182	*	0.0882	0.7715	-
A^2^	2.11	0.1717	-	10.12	0.0079	**
B^2^	17.89	0.0012	**	7.01	0.0213	*
C^2^	20.43	0.0007	**	11.06	0.0060	**
D^2^	1.49	0.2460	-	2.46	0.1431	-
Residues	33.22	0.0296	-	4.46	0.1970	-

Note: 0.01 < *p* < 0.05, the difference is significant, indicated by ‘*’. *p* < 0.01, the difference is extremely significant; that is, the impact on the research indicator is more obvious, indicated by ‘**’. *p* > 0.05, not significant, indicated by ‘-’.

**Table 2:** Factors and associated levels.

Factor	Variables	Unit	Levels		
			Low	Medium	High
A	Zn(NO_3_)_2_	mol/L	0.025	0.050	0.075
B	HMTA	mol/L	0.050	0.075	0.100
C	Temperature	°C	85	90	95
D	Duration	h	1	2	3

## Data Availability

Data are contained within the article.

## Supplementary Materials

The following supporting information can be downloaded at: https://www.mdpi.com/article/10.3390/molecules29122916/s1, Table S1: ZNRN antibacterial capabilities in *E. coli* and *S. aureus* of individual factorial combinations; Table S2: Fitting information; Figure S1: EDS pattern of ZNRN; Figure S2: XPS pattern of ZNRN; Figure S3: SEM and observation pictures; Figure S4: SEM and TEM; Figure S5: FC.
