# Supplementary material for: In Situ Self-Growth of a ZnO Nanorod Array on Nonwoven Fabrics for Empowering Superhydrophobic and Antibacterial Features
Source: Molecules. 2024 Jun 19;29(12):2916. doi: 10.3390/molecules29122916 (PMC11206326; doi:10.3390/molecules29122916)
Supplement: Supplementary file 1 [file molecules-29-02916-s001.zip › molecules-2978148-supplementary.pdf]

Supporting Materials

1. Experimental design based on response surface methodology

In order to identify the desired superhydrophobic and antibacterial capabilities of ZNRN, a total of 27 combinations (three levels of four factors) were proposed based on Box-Behnken design (BBD). The detail of these 27 combinations was listed in Table S1. In addition, a second-order regression model (quadratic model) was expected to be fitted for analyze the correlation ships between the features and synthesis factors and the resultant fitting information was described in Table S2. The BBD was undertaken through Design Expert at a significance level of 0.05.

Table S1 ZNRN antibacterial capabilities in *E. coli* and *S. aureus* of individual factorial combinations

Std	Run	ZnO /(mol/L)	HMTA/(mol/L)	Temperature /℃	Duration /h	*E. coli* antibacterial rate /%	*S. aureus* antibacterial rate /%
26	1	0.05	0.075	90	2	83.8	79.64
25	2	0.05	0.075	90	2	99.79	81.67
7	3	0.05	0.075	85	3	97.61	74.35
15	4	0.05	0.05	95	2	40.94	62.6
11	5	0.025	0.075	90	3	99.68	61.03
27	6	0.05	0.075	90	2	99.65	83.91
2	7	0.075	0.05	90	2	40.23	63.27
23	8	0.05	0.05	90	3	73.76	61.81
8	9	0.05	0.075	95	3	65.52	62.71
10	10	0.075	0.075	90	1	59.55	74.18
13	11	0.05	0.05	85	2	65.51	60.92
1	12	0.025	0.05	90	2	75.81	61.55
9	13	0.025	0.075	90	1	97.26	76.17
24	14	0.05	0.1	90	3	99.65	75.01
4	15	0.075	0.1	90	2	73.03	80.59
3	16	0.025	0.1	90	2	76.4	81.29
22	17	0.05	0.1	90	1	45.68	83.25
12	18	0.075	0.075	90	3	69.42	78.43
16	19	0.05	0.1	95	2	75.81	75.28
17	20	0.025	0.075	85	2	88.2	66.24
6	21	0.05	0.075	95	1	65.38	73.66
14	22	0.05	0.1	85	2	33.71	82.08
5	23	0.05	0.075	85	1	41.96	82.8
20	24	0.075	0.075	95	2	87.18	69.93
19	25	0.025	0.075	95	2	48.46	73.71
21	26	0.05	0.05	90	1	65.52	76.48
18	27	0.075	0.075	85	2	51.53	69.95

Table S2 Fitting information

Item	Statistical Values	
	Antibacterial Rate of *E. coli*	Antibacterial Rate of *S. aureus*
R^2 (Coefficient of Determination)	0.8832	0.8697
R^2adj (Adjusted Coefficient of Determination)	0.7469	0.7178
CV (Coefficient of Variation)	14.23%	5.76%
Adeq Precision (Signal-to-Noise Ratio)	8.1907	7.6546

2. Surface chemical composition analysis of ZNRN

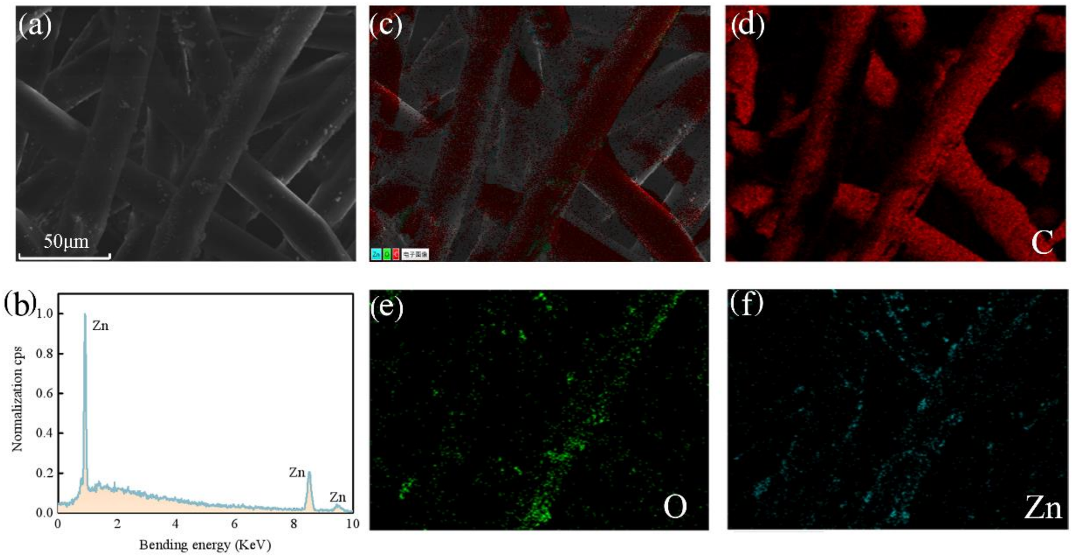

Figure S1 EDS pattern of ZNRN.

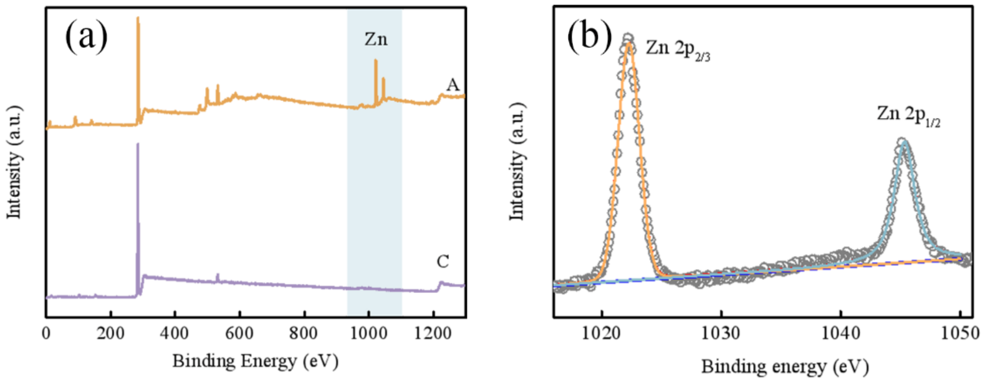

Figure S2 XPS pattern of ZNRN.

3. Observation of antibacterial performance of ZNRN

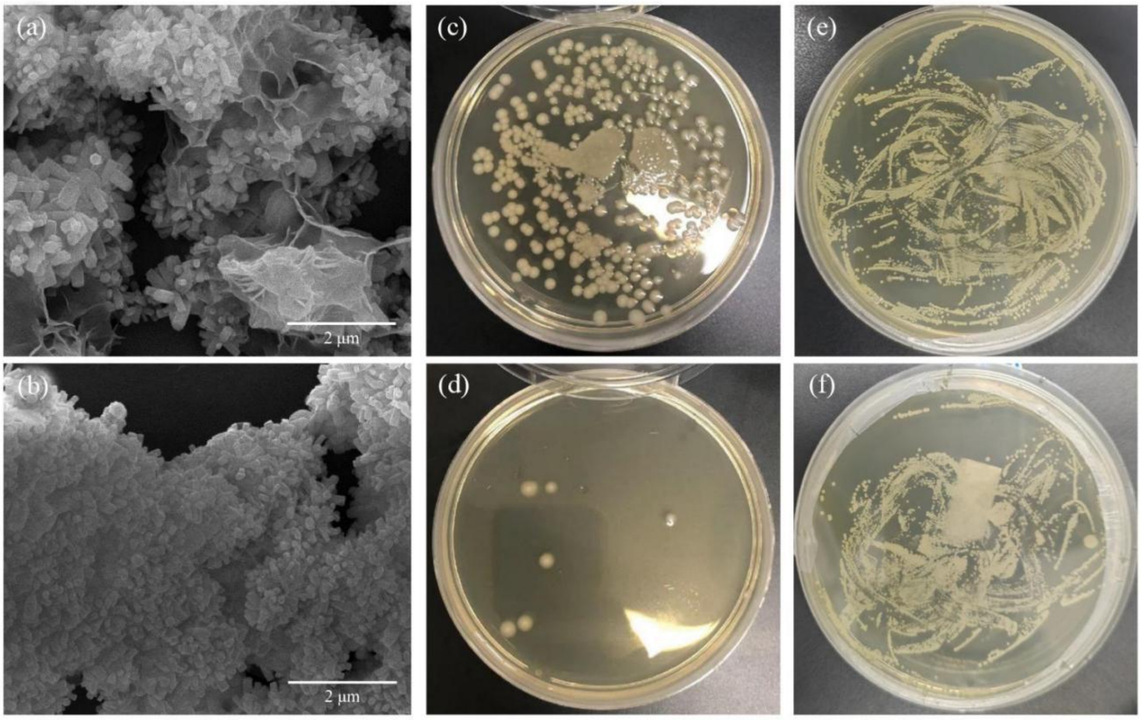

Figure S3 SEM and observation pictures. (a) and (b) are SEM images of non-optimized and optimized ZNRN samples, respectively; (c) and (e) are optical pictures of survival *E. coli* and *S. aureus* colonies after contacted with non-optimized ZNRN, respectively; (d) and (f) are optical images of survival *E. coli* and *S. aureus* colonies after contacted with optimized ZNRN, respectively.

4. Verification of antibacterial mechanism of ZNRN

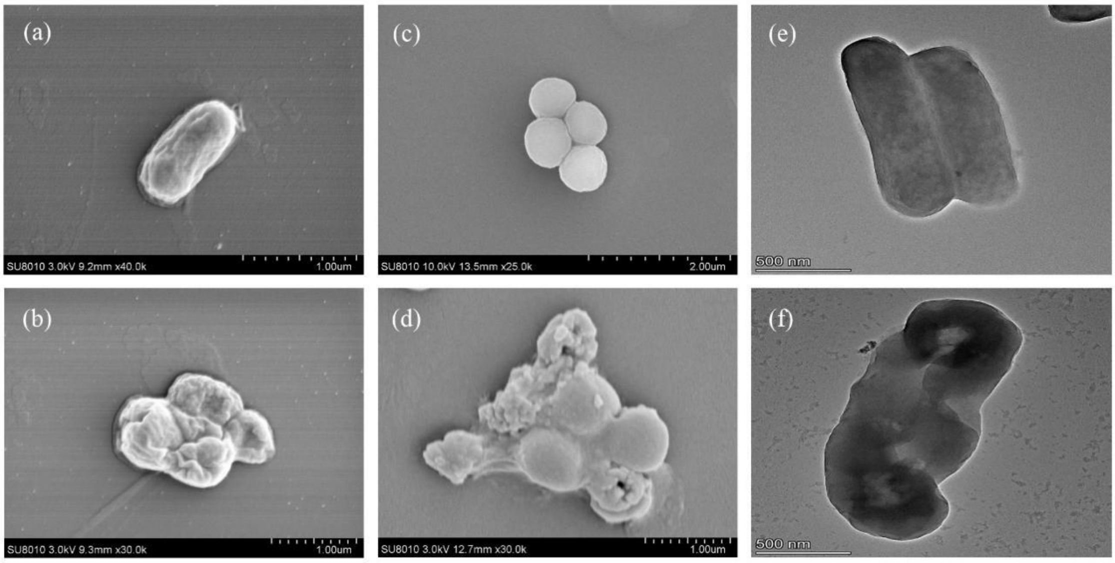

Figure S4 SEM and TEM. (a) and (b) are the original morphology of *E. coli* under SEM and the morphology of cell membrane/wall damage after *E. coli* contact with ZNRN, respectively. (c) and (e) are the original morphology of *S. aureus* under SEM and the damaged morphology of cell membrane/wall after *S. aureus* contact with ZNRN, respectively. (d) and (f) are the original morphology of *E. coli* under TEM and the morphology of cell contents leakage after *E. coli* contact with ZNRN, respectively.

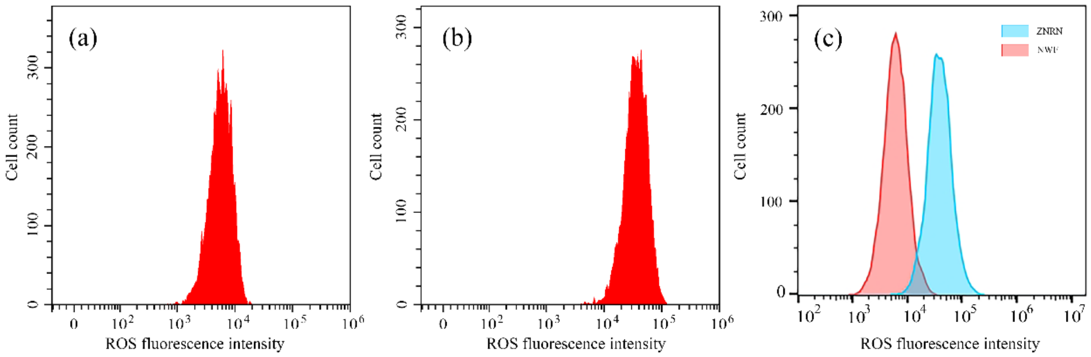

Figure S5 FC. (a) is the fluorescence value of ROS measured after 30min contact between *E. coli* and NWF (non-woven fabrics); and (b) is the fluorescence value of ROS measured after 30min contact between *E. coli* and ZNRN; and (c) is the fitting comparison diagram.
